# Particulate air pollution and impaired lung function

**DOI:** 10.12688/f1000research.7108.1

**Published:** 2016-02-22

**Authors:** Laura Paulin, Nadia Hansel

**Affiliations:** 1 Division of Pulmonary and Critical Care, Johns Hopkins School of Medicine, Baltimore, Maryland, 21224-6801, USA

**Keywords:** Air pollution, Particulate matter, Lung function, asthma, COPD, lung development

## Abstract

Air pollution is a leading cause of morbidity and mortality throughout the world, particularly in individuals with existing lung disease. Of the most common air pollutants, particulate matter (PM) is associated with an increased risk of exacerbations and respiratory symptoms in individuals with existing lung disease, and to a lesser extent, in those without known respiratory issues. The majority of published research has focused on the effects of PM exposures on symptoms and health care utilization. Fewer studies focus on the impact of PM on objective measurements of pulmonary function. This review will focus on the effects of PM exposure on objective measurements of lung function in both healthy individuals and those with existing lung disease.

## Introduction

Air pollution is associated with millions of premature deaths worldwide ^1^, 25% of which are estimated to be respiratory in nature ^2^, and is the world’s largest environmental health risk ^3^. In an attempt to mitigate ambient exposure to air pollution, the Environmental Protection Agency (EPA) currently regulates six criteria air pollutants under the Clean Air Act ^4^. Of the regulated pollutants, particulate matter (PM) has been extensively studied and associated with a myriad of adverse health outcomes, including an adverse impact on lung function in both children and adults ^5– 7^. PM is a complex mixture of liquid droplets and extremely small particles composed of organic and inorganic compounds ^7^. PM less than 10 μg in aerodynamic diameter, designated by PM _10_, can penetrate conducting airways; PM _2.5_ (PM less than 2.5 μg in aerodynamic diameter) is composed of fine particles and can penetrate into the gas-exchanging regions of the lung ^6^. Sources of ambient PM include construction sites, smokestacks, fires, power plants, and automobiles; the main sources of indoor PM include ambient PM, tobacco smoke, cooking, and heating appliances. PM causes lung inflammation and mucous secretion by acting on airway epithelial cells and alveolar macrophages and may lead to airway remodeling ^8^.

Obstructive lung diseases, including chronic obstructive pulmonary disease (COPD) and asthma, are a large source of morbidity and mortality across the entire world ^9, 10^. Exposure to PM has been associated with worse morbidity and mortality in patients with COPD and asthma, including worse symptoms and quality of life and higher exacerbation rates and health care utilization ^11– 17^. Lung function is often used as an indicator of disease severity, and several studies have also focused on the impact of PM exposure on objective measures of lung function. This review will focus on the health effects of PM exposure on lung function in healthy children and adults, and in those with existing pulmonary disease.

## Impact of PM exposure *in utero* and during infancy

Children may be particularly sensitive to the health effects of PM exposure, and early exposure during critical time points in lung development may lead to lasting impacts on lung function ^18– 20^. Several studies show that exposure to PM *in utero* is associated with decreased lung function in children, suggesting that fetal lung growth is impacted by maternal PM exposures ^21^. For example, in a Californian study of 232 children with asthma, fetal exposure to ambient PM _10_ during the first trimester of pregnancy was associated with a lower peak expiratory flow (PEF) between the ages of 6 and 11 ^22^. In a study of 176 children of non-smoking mothers, Jedrychowski *et al.* measured maternal exposure to PM using a backpack worn for 48 hours and showed that children exposed to higher concentrations of PM _2.5_ *in utero* had an approximately 90 mL lower forced expiratory volume in 1 second (FEV _1_) at 5 years of follow-up ^23^. Exposure to higher concentrations of PM _10_ during the first year of life was associated with a FEV _1_ reduction of nearly 60 mL in Swedish children aged 8 years old, suggesting that exposures during infancy have lasting health effects years later ^24^. Importantly, analysis of the same cohort at 16 years of age shows that children with exposure to higher concentrations of PM _10_ during the first year of life (above the median concentration) were more likely to have FEV _1_ and forced vital capacity (FVC) less than the lower limit of normal ^25^. While these studies suggest *in utero* and infancy PM exposure leads to lower lung function, others have not supported the relationship between early PM exposure and lung function decrements. In a German study of 2226 children, investigators used comprehensive land-use regression modeling to estimate yearly average PM concentrations at the time of each participant’s birth. Early long-term PM exposure was not found to be associated with spirometric outcomes at 15 years of age ^26^. In a Swiss study, maternal exposure to PM _10_ measured daily during pregnancy was not found to be associated with lung volumes in 241 healthy newborns ^27^. To address the knowledge gaps that persist, large cohort studies with frequent comprehensive participant evaluations and individual-level PM exposure assessments are necessary to identify potential time points of increased susceptibility of the developing lung.

## Impact of PM exposure in healthy children

The research evaluating the impact of PM exposure on lung function in healthy children is relatively scarce and inconsistent. Several observational studies demonstrate that PM exposure may have short- and long-term impacts on lung function in children without known lung disease. A longitudinal study by Castro *et al.* found that an increase in 2-day lagged PM was associated with a 0.34 L/min decrease in PEF in 118 schoolchildren in Rio de Janeiro ^28^. Similarly, an air pollution episode resulting in a 1-day PM _10_ concentration of 174 μg/m ^3^ was associated with worse same-day spirometry in 112 healthy Dutch children ^29^. Higher concentration of 8-hour PM, measured on the morning prior to lung function testing, was associated with a marginal decrease in FEV _1_ and FVC in 163 healthy Austrian schoolchildren ^30^. However, after accounting for nitrogen dioxide concentrations, this association was attenuated, highlighting the importance of identifying all exposures that may impact lung function.

The Children’s Health Study followed respiratory growth over a period of 8 years in 1759 Californian children. Children exposed to the highest PM _2.5_ concentrations had an approximately 80 mL lower FEV _1_ than children exposed to the lowest level of PM ^19^, an effect size similar to observed values in children exposed to maternal smoking ^19, 31, 32^. In the same cohort, children living in residences closer to roadways, with higher estimated PM concentrations, had approximately 3% lower FEV _1_^33^, highlighting the contribution of traffic-related pollution to respiratory health. A study of 2307 healthy 10-year-old children in Norway found that an increase in the lifetime exposure to PM was associated with a 58–66 mL/sec lower PEF ^34^, and in 179 British schoolchildren followed for several months, higher PM was associated with a 4% increase in the odds of having a lower PEF (defined as greater than 20% below the median) ^35^.

There were no consistent associations between PM exposure and lung function in a 3-year longitudinal study of 1150 Austrian schoolchildren, including both healthy children and those with asthma ^36^. In an analysis of data from the Six Cities Study, indoor measurements of PM _2.5_ showed no direct association with pulmonary function measurements in a random sample of 2994 children ^37^. The discrepancy in research may be due to difficulty in performing lung function testing in children, or in part due to the nature of the exposure and unmeasured changes in PM concentration over time. Although there are few cohorts that extend from childhood to adulthood, these findings suggest that exposure in early life may have implications in the development of future airway disease and highlight the need for comprehensive longitudinal studies.

## Impact of PM exposure in children with asthma

Children with existing lung disease are likely to be more susceptible to lung function changes following exposure to PM. In a study of 326 elementary schoolchildren in Seattle, 24 of whom had asthma, a 20 μg/m ^3^ increase in ambient PM _2.5_ concentration measured over 2 years at the children’s school was associated with a 34 and 37 mL drop in FEV _1_ and FVC, respectively, in those with asthma ^38^. British children with wheezing had increased odds of having a drop in PEF when exposed to higher concentrations of outdoor PM measured over the course of a winter season ^35^. Exposure to higher PM concentrations over shorter time periods can lead to changes in lung function as well. When exposed to higher concentrations of ambient PM _2.5_ over a 3-day period, children with asthma in Canada were found to have a decrease in FEV _1_ and FVC ^39^. An increase of 10 μg/m ^3^ in 24-hour mean PM was associated with a drop of 3 L/min in PEF in Japanese children hospitalized for severe asthma exacerbations ^40^. Delfino *et al.* followed 53 children with asthma living in Los Angeles and found that an interquartile range (IQR) increase in 1-hour peak PM was associated with a 1.32% decrease in same-day percent predicted FEV _1_^41^. In a study of 19 children with asthma, Allen *et al.* found that a higher 24-hour concentration of PM _2.5_, measured over a period of 10 days, was associated with worse lung function as measured by daily PEF and FEV _1_^42^. Although less studied, short-term increases in indoor PM exposures have also been associated with lower lung function in children with asthma. A higher 24-hour indoor PM concentration measured over a period of 10 days was associated with a decrease in same-day and 1-day lagged PEF and FEV _1_ in Seattle children with asthma ^43^. In another study, higher 24-hour indoor PM concentrations were associated with worse PEF in 22 children with asthma living in the United Kingdom ^44^.

Studies examining the relationship between PM exposure and lung function in children with asthma have been inconsistent. In the Children’s Health Study, there was no significant effect of PM exposure on lung function in the 483 children with a history of doctor-diagnosed asthma ^19^. The authors suggest that the smaller sample size in this subgroup may have contributed to these findings. Noting the lack of consistency in quantitative outcomes, Weinmayr *et al.* completed a meta-analysis of 36 studies evaluating the association of PM and respiratory symptoms and PEF in children with asthma or asthma-like symptoms. Although there is clear evidence of worsening respiratory symptoms associated with higher PM concentrations, the authors report a non-significant decrease in PEF of 0.082 L/min per unit increase in PM ^45^. Furthermore, investigators have often noticed a disconnect between asthma symptoms and lung function ^46^, suggesting that lung function changes may not be the most sensitive marker of disease burden in children.

## Impact of PM exposure on adults

Although the developing lung may be more vulnerable to PM exposures, several studies show that adults are susceptible to air pollution exposures as well. The SAPALDIA study of nearly 10,000 Swiss adults showed that a 10 μg/m ^3^ increase in PM _10_ was associated with a 3.4% decrease in FVC in a cross-sectional analysis, even after adjusting for cigarette use ^47, 48^. In the Normative Aging Study, 858 elderly men living in the Boston area were followed for a period of over 10 years, and exposure to ambient black carbon (a marker of traffic-related PM) was estimated using spatiotemporal land use regression models. Higher yearly black carbon concentration was associated with an increase in the normal age-related rate of decline of FEV _1_ and FVC ^49^. Similarly, higher concentrations of previous-day ambient PM _2.5_ were associated with approximately 20 mL lower FEV _1_ and FVC in nonsmoking adults in the Framingham Heart study ^50^, and exposure to higher long-term concentrations of PM (yearly levels) was associated with lower FEV _1_ and a faster rate of lung function decline ^51^. In this cohort, followed for over 10 years, a 10 μg/m ^3^ increase in PM _2.5_ was associated with an additional 10.5 mL annual decline in FEV _1_ beyond the normal age-related decline, suggesting that exposure to even moderate concentrations of PM can lead to meaningful lung function decline in healthy adults. Though the absolute changes in lung function in the above studies are of relatively low magnitude, prior research has shown that decreases in FEV _1_ are associated with all-cause mortality ^52, 53^ and that lower lung function is associated with higher rates of cardiovascular disease ^54, 55^.

## Adults with existing lung disease

There are few studies investigating the effects of PM on lung function in adults with established lung disease, such as COPD and asthma. The studies that do exist suggest that among individuals with established COPD, long-term exposure to outdoor PM may be linked to lung function decline. For example, PM _2.5_ exposure was associated with FEV _1_ decline among 1218 subjects with severe COPD followed for an average of 29.2 months in the National Emphysema Treatment Trial (NETT) ^56^. Similarly, among 401 individuals with COPD and α-1-antitrypsin deficiency, a 10 µg/m ^3^ increase in PM _10_ was associated with an additional 30 mL/year decline in FEV _1_^57^. Though these studies are suggestive, whether these findings extend to patients without α-1-antitrypsin deficiency or severe emphysema is unclear. Long-term studies of air pollution on a diverse group of subjects with COPD, including those with heavy smoking exposure, are needed to further clarify the effects of air pollution on COPD progression.

Short-term variation in pollution may also be associated with changes in lung function in patients with chronic lung disease; however, studies investigating short-term variation in pollution and lung function in adults with COPD or asthma have been inconsistent and interpretations are limited by small sample sizes. For example, Peacock *et al.* recruited 94 COPD subjects who filled out daily diaries. An IQR increase in PM _10_ was linked to an approximately 13% increase in odds of symptomatic decreases in PEF, defined as a fall in PEF for at least 2 days plus a reported increase in dyspnea ^11^. In a recent Italian study, increased outdoor PM _10_ concentrations were associated with lower FEV _1_ and FVC in COPD patients presenting with urgent hospitalization ^58^. Similarly, Lagorio *et al.* showed that increased ambient PM _2.5_ and PM _10_ concentrations were associated with lower lung function (FEV _1_ and FVC) in subjects with COPD, and the effect on FEV _1_ appeared only when 72 hours of exposure were accumulated ^59^. However, other studies have not shown a link between PM and short-term changes in lung function among those with COPD. A small panel study including 17 subjects with COPD showed no consistent association between PM with lung function over 12 days ^60^. A cohort of patients with COPD followed for 3 months found that an increase in PM _10_ was associated with an increase in nighttime symptoms, but there was no change in lung function ^61^. Similarly, Hansel *et al.* identified that indoor PM is linked to respiratory symptoms and exacerbations, but not lung function, among former smokers with COPD ^15^.

Studies that have investigated the short-term effects of PM on lung function in subjects with asthma have also been inconsistent. In a real-time exposure study of 60 adults with mild to moderate asthma, McCreanor *et al.* found that FEV _1_ decreased up to 6.1% following a 2-hour walk in a high-traffic area (with corresponding higher PM _2.5_ concentration) as compared to a walk in a park (with lower PM _2.5_ concentration) of similar duration ^62^. In a longitudinal analysis, Park *et al.* found an increase in PM _10_ concentration was associated with increases in PEF variability of >20% and a decrease in the mean PEF among 64 adults with asthma ^63^. Balmes *et al.* noted that those with asthma with the middle and highest tertiles of ambient PM _2.5_ exposure had an increased risk of having FEV _1_ below the lower limit of normal (odds ratio [OR]=1.93 95% confidence interval [CI]: 0.94, 3.95 and OR=2.23 95% CI: 1.08, 4.61), but only among females ^64^. Using biweekly spirometry over 6 months on a group of 54 adult asthmatics, Penttinen *et al.* found that particle number concentrations on the preceding days were inversely, but mostly non-significantly, associated with FEV _1_, FVC, and PEF, and no associations were observed with larger particles (PM _10_) ^65^. Several other studies showed no association between PM and lung function in adults with asthma. Though Lagorio *et al.* had observed a negative association between PM and lung function in subjects with COPD, there was no association between PM and lung function decrement among those with asthma ^59^. Another study including subjects with COPD and asthma showed no association between indoor or outdoor air quality on lung function ^66^. A study from Italy by Maestrelli *et al.* followed adult subjects with asthma periodically over 2 years and showed that measured personal exposure to PM _10_ during the 24 hours prior to assessment was associated with respiratory symptoms, but not FEV _1_^67^.

Given the relatively small size of studies of air pollution on short-term changes in lung function in adults with COPD and asthma, they may not have been adequately powered to detect an association between pollutant exposure and lung function. Alternatively, it is possible that the adverse effects of PM exposure are linked to changes in smaller caliber airways that are not adequately captured by spirometric measures such as FEV _1_.

## Effect of PM reduction on lung function

While the observational findings linking higher PM to worse lung function are intriguing, there is a need to provide additional levels of evidence, including assessment of temporality and observation as to whether lung function improves with reduction of PM exposure, to establish causality. Importantly, studies have shown that a decrease in pollution can improve lung function in both children and adults. For example, the Children’s Health Study in southern California followed three separate cohorts of children during periods of increasing PM regulation and therefore decreasing average PM concentrations. Investigators found higher FEV _1_ and FVC in the children exposed to the lowest concentrations of PM and, as air quality improved, the proportion of children with clinically low FEV _1_ subsequently declined ^68^. Similarly, reductions in PM in an urban area of the Netherlands were associated with a 3% and 6% improvement in FEV _1_ and FVC, respectively, in both children and adults ^69^. In the SAPALDIA study, a 10 μg/m ^3^ decrease in PM _10_ concentration was associated with a 9% decrease in the rate of yearly decline of FEV _1_ in over 9000 randomly selected Swiss adults ^70^. These studies highlight that even modest reductions in PM can translate to meaningful improvements in lung function.

As the EPA does not regulate the indoor environment, interventions to mitigate exposure to indoor PM are less well studied. A small number of research studies have shown improvements in indoor PM concentrations following behavioral modifications and use of indoor air cleaners ^71– 73^. Most of these studies did not assess the impact of these modifications on lung function, and the results are inconsistent in the ones that do. In one study of 48 nonsmoking adults, air cleaners placed in the living room and bedroom decreased PM _2.5_ concentrations but did not improve lung function in these participants, although conclusions may be limited by the small sample size ^74^. In a randomized study of active versus sham air cleaners in 35 healthy college students in China, 48 hours of air purification significantly reduced indoor PM _2.5_ concentration by 57%. This decrease was associated with a significant reduction in circulating inflammatory markers, along with a non-significant trend towards slight improvement in lung function following air purification ^75^. Air cleaners that were run for 1 week in 20 homes on the First Nations reserve in Canada significantly decreased indoor PM _2.5_ concentrations, a reduction that was associated with a 217 mL increase in FEV _1_ in 37 healthy individuals living in the homes ^76^. These studies highlight the potential for indoor air modification to reduce total PM exposure; whether the improvement in indoor air quality can lead to improvements in lung function is yet to be determined.

## Potential mechanisms of susceptibility to PM

The precise mechanism as to how PM may influence health and lung function is unknown. Studies have suggested that PM may mediate adverse health effects via the generation of reactive oxygen species ^77– 79^, activation of cell signaling pathways, and alterations of respiratory tract barrier function and antioxidant defenses, all of which may lead to airway inflammation and changes in pulmonary function ^80^. Additionally, cellular changes resulting from PM exposure may cause epigenetic modifications, leading to alterations in gene expression ^81^. For example, results from the ENVIRONAGE birth cohort showed that PM _2.5_ exposure during gestation was associated with placental mitochondrial DNA methylation in 381 mother-newborn pairs ^82^. These studies may offer insight into how PM exposure *in utero* may impact lung function later in life. Furthermore, activation of many cellular signaling pathways has been attributed to specific chemical and metal constituents of PM that have been isolated *in vitro*. This suggests that PM of various sources may lead to diverse responses, making compositional analysis of PM by region an important consideration for future research ^81, 83^.

Patient factors, including demographic and genetic factors, may modify the impact of PM exposures on lung function. Our review reinforces the conclusion that PM exposure can have important impacts on lung function in those with and without existing lung disease; however, whether individuals with pre-existing respiratory disease are more susceptible to the adverse effects of PM exposure is unclear. Few studies examine the variability in lung function response to PM exposure specifically by respiratory disease status. Toxicological studies suggest that the presence of allergic airway conditions may increase susceptibility to PM exposure; however, epidemiologic studies have been inconsistent in clarifying this relationship ^84^. For example, in Koenig *et al.*’s study of Seattle schoolchildren, PM exposure impacted lung function in only those children with asthma, not healthy children ^38^. Conversely, in a study of Mexico City schoolchildren, decrements in FVC following higher exposure to PM _2.5_ were seen in both the 158 asthmatic children and the 50 children without asthma ^85^. Similarly, individuals with COPD may have impairment in mucociliary clearance, which may lead to an increase in dose of fine particles ^86^ and resultant greater risk of PM-related respiratory effects, although epidemiological studies are needed to confirm this hypothesis ^84^. Additionally, several studies have suggested that the impact of PM on lung function may vary by gender, and early life may be a critical time window when PM may adversely affect lung function. A few studies have also investigated whether genetic polymorphisms may modify the effects of PM on lung function. For example, results from Breton *et al.* suggest that genetic variations in the glutathione synthesis pathway may modify the impact of PM exposure on lung function in children ^87^ and several investigations from the SAPALDIA cohort suggest that genetic variations potentially mediate the effect of PM on lung function decline ^88, 89^. Identifying risk factors of those who will be more susceptible to the health effects of PM exposure is a research priority.

## Conclusion

In summary, the existing research suggests that PM exposure can influence lung development and have an important impact on lung function in both children and adults, and in those with and without existing lung disease. Although not without inconsistencies, this research adds to the wider body of literature that supports an association between PM exposure and worse respiratory symptoms. To address the knowledge gaps that persist, large cohort studies with frequent comprehensive participant evaluations and individual-level PM exposure assessments are needed. In particular, studies on indoor air pollution have tended to be small, making definitive conclusions more challenging. In addition, size and composition of PM may have differing health effects; however, these differential effects have not yet been clearly elucidated. Lastly, there are several factors that may mediate the effects of PM exposure on lung health, such as timing of exposure, chronic lung diseases (including but not limited to obstructive airway diseases), genetics, and even other exposures such as medication use. Importantly, the studies to date suggest that relatively low concentrations of PM can negatively impact lung function, suggesting that meaningful health outcomes occur following exposure to relatively modest pollutant concentrations, many of which fall below the current EPA limits. Furthermore, while the observed decrements in lung function are relatively small, they have the potential to be clinically meaningful, especially in children, as those with lower lung function have been shown to have an increased risk of developing asthma ^90^.

Moreover, the improvement in lung function seen following PM reduction implies that the majority of the population will benefit from continued regulation of PM levels. In addition, the importance of short-term exposures is reflected in EPA standards that regulate not only average annual PM concentration but also 24-hour concentrations. To reflect the growing body of evidence suggesting the adverse impact of short-term PM exposures, these standards were strengthened in 2006 ^6^. Policies and methods to decrease PM exposure are crucial to minimize the health impact of continued PM exposure on lung health. Due to the regulatory programs delineated as part of the original 1970 Clean Air Act and the 1990 amendments, there has been a 34% decrease in the annual national average of PM ^91^. It is hoped that continued regulation of PM will help alleviate the impact that exposure has on the developing and adult lung.
